# Selective impairments of alerting and executive control in HIV-infected patients: evidence from attention network test

**DOI:** 10.1186/s12993-017-0129-0

**Published:** 2017-06-27

**Authors:** Yi-quan Wang, Yang Pan, Sheng Zhu, Yong-guang Wang, Zhi-hua Shen, Kai Wang

**Affiliations:** 1 Department of Brain Functioning Research, The Seventh Hospital of Hangzhou, 305 Tianmushan Road, Hangzhou, 310013 Zhejiang China; 2 Clinical Institute of Mental Health in Hangzhou, Anhui Medical University, Hangzhou, Zhejiang China; 3 School of Media and Design, Hangzhou Dianzi University, Hangzhou, Zhejiang China; 4 The Fifth Hospital of Ruian, Ruian, Zhejiang China; 5 Collaborative Innovation Center for Neuropsychiatric Disorders and Mental Health, Hefei, Anhui China; 6 Department of Neurology, The First Affiliated Hospital of Anhui Medical University, Hefei, China

**Keywords:** HIV, Attention network test, Alerting, Orienting, Executive

## Abstract

**Background:**

Attention ability can be subdivided into three functionally independent networks, i.e., alerting network, orienting network, and executive network. Previous literature has documented that deficits in attention are a common consequence of HIV infection. However, the precise nature of deficits of attention in HIV-infected patients is poorly understood. Accordingly, the aim of the study was to identify whether the HIV-infected patients showed a specific attention network deficit or a general attentional impairment.

**Methods:**

We investigated 27 HIV-infected patients and 31 normal controls with the Attention Network Test (ANT).

**Results:**

The patients exhibited less efficient alerting network and executive network than controls. No significant difference was found in orienting network effect between groups. Our results also indicate a tendency for poorer efficiency on alerting attention and executive attention in patients with CD4 ≤ 200.

**Conclusions:**

Our findings suggest that HIV-infected patients exhibited selective impairments of attention network of alerting and executive control. The link between lower CD4 T cell count and poorer attention network function imply the importance of starting antiretroviral therapy earlier to avoid irreversible neurocognitive impairment.

## Background

HIV-associated neurocognitive disorders (HAND) are characterized by disabling cognitive, behavioral and motor dysfunction [1], and are a common hallmark of HIV infected individuals. Although the introduction of combination antiretroviral treatment (cART) has significantly reduced the prevalence of more severe form of HAND [2, 3], the incidence of less severe forms of HAND (i.e., asymptomatic neurocognitive impairment and mild neurocognitive disorder) remain common among HIV patients in the cART era [3–5]. Given the predominance of the milder forms of HAND [4, 6, 7], considerable researches have been conducted to elucidate the component processes of HAND across the domains of information processing speed, executive functions, motor skills, episodic memory, and etc. [8]. Additionally, neuroimaging studies have found HIV-infected patients exhibited hyperactivity in task-related brain regions despite equal performances as controls [9, 10], suggesting that functional compensation by increasing usage of neural reserves to maintain cognitive performance.

Attention is a core property in human information processing [11], which enables us to process behaviorally relevant information for the guidance of our responses [12]. For HIV-infected patients, deficits in attention are a common consequence of HIV infection [13, 14] and the one of the neurocognitive domains affected early in progression of HIV [14, 15]. Although attention plays the central role in patients’ driving ability [16] and was associated with poor medical adherence [17], the precise nature of deficits in attention for HIV-infected patients is poorly understood [14]. In previous studies of HAND, attentional functioning was routinely investigated by clinical neuropsychological tests. While the neuropsychological approach has its clinical convenience, it also has two main disadvantages. Firstly, although these studies considered attention as one of a number of distinguishable cognitive domains, the neuropsychological tests using in these researches required multiple cognitive abilities for successful performance [14]. Secondly, this approach pertained merely to matters of overall attentional functioning rather than the structures of attention. The lack of consistency in the definition of attention [13] would make it difficult to compare results across studies.

Conceptually, attention is not a single entity, but is comprised of multiple components. According to the attention network theory, this basic and sophisticated cognitive ability can be subdivided into three functionally independent networks, i.e., alerting network, orienting network, and executive network [18, 19]. In this frame, the alerting network allows maintenance of a vigilant and alert state, the orienting network allows for selecting the information through the space, and the executive network is responsible for solving the conflict between expectation, stimulus, and response [18].

The Attention Network Test (ANT) was developed to assess the ability of these three separate networks. The efficiency of each network is calculated by averaging reaction times across several different cue and flanker conditions. Since the initial description of the ANT [20], attention network function has been examined using the ANT in different clinical population, including schizophrenia [21], depression [22], ADHD [23], 22q11 deletion syndrome [24], multiple sclerosis [25], and etc. These studies suggested that most of these neuropsychiatric populations exhibited a specific attention network deficit rather than a general attention deficit.

As described above, although deficits in attention are deemed as the one of cognitive declines among HIV-infected patients, however, previous studies failed to address whether the HIV-infected patients showed a specific attention network deficit or a general attentional impairment. Accordingly, we examined HIV-infected patients with the attention network test, to better understand the nature of attentional deficits in the population.

## Methods

### Participants and procedures

A total of twenty-seven patients with HIV-1 infection were recruited through the voluntary counseling and testing clinic at Hangzhou Center for Disease Control and Prevention. Among these patients, there were thirteen patients were diagnosed with AIDS, according to the clinical history of AIDS defining illnesses or the nadir CD4 T cell count below 200 cells per microliter. In addition, there were twenty patients with nadir CD4 T cell count below 400 per microliter, including twelve patients being treated with highly active antiretroviral therapies (HAART) containing a HIV protease inhibitor and eight patients that refused HAART therapy.

The average nadir CD4 T cell count for all patients was 298.41 (SD = 223.58) cells per microliter. According to the previous literature [26], to study the possible association between a nadir CD4 T cell count and the pattern of attention network function, all patients were divided into patients with CD4 ≤ 200 (n = 12) and patients with CD4 > 200 (n = 15).

Thirty-one healthy controls with no HIV-infection history were recruited from local community to serve as a control group. All participants were interviewed by an experienced clinical psychologist with the Structured Clinical Interview for DSM-IV (SCID) [27] and met the following inclusion criteria: (a) negative family history of any psychiatric disorders, (b) no evidence of current or previous head injury, CNS disease or DSM-IV Axis I disorder, (c) no evidence of current alcohol or substance abuse, and (d) no evidence for severe opportunistic infections. All participants were over 18 years of age and right-handed, with normal vision and hearing. Written and informed consent was obtained from all participants. The details of participants were shown in Table 1.

**Table 1:** Comparisons of demographic between groups (mean ± SD)

	Normal controls (n = 31)	HIV-infected patients (n = 27)	Statistics
Sex ratio (M: F)	18:13	17:10	*χ* ^2^= 0.145, *p* = 0.704
Index age (years)	30.97 ± 12.83 (16–60)	32.26 ± 7.31 (21–45)	*F* = 0.213, *p* = 0.646
Education levels (years)	11.81 ± 4.42 (4–17)	10.48 ± 4.64 (4–18)	*F* = 1.241, *p* = 0.270

### ANT

**Fig. 1:**
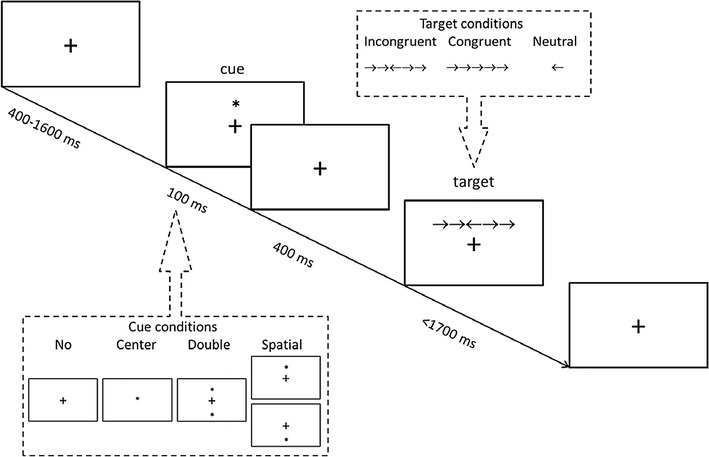
The diagram of attention network test

The details of ANT [20] are illustrated in Fig. 1. At the beginning of each trial, a fixation cross was presented in the center of the screen for a random variable duration (400–1600 ms). Then, a warning cue was presented for 100 ms. After a short fixation period (400 ms), the target appeared and participants were required to determine whether a central arrow point left or right. Participants were required to focus on centrally located fixation cross throughout the task, and to response by pressing the keyboard direction key as fast and accurately as possible.

There are four different cue conditions: (1) no cue, participants were shown a fixation cross which was the same as the first fixation across; (2) center cue, in which an asterisk was presented at the central fixation across; (3) double cue, in which an asterisk was presented on above and below the fixation cross, separately and simultaneously; (4) spatial cue, in which an asterisk was presented on the target location (either above or below the central fixation point). Also, there are three target conditions: (1) neutral target, there was only one central arrow; (2) congruent target, the target (i.e., central arrow) was flanked on either side by two arrows in the same direction; (3) incongruent target, the target was flanked on either side by two arrows in the opposite direction.

Each trial lasted for 4000 ms. In total, there were one practice block and three experimental blocks. The practice block consisted of 24 trials with feedback on their speed and accuracy. Each experimental block consisted of 96 trials without feedback.

### Calculation of attention network efficiencies

The manipulations of cue and target allow the calculation of response time (RT) difference assumed to represent the three attention networks. According to previous literature [21, 28], to avoid the influence of the outliers, wrong responses, RT outside a 100–1700 ms window and RT outside two standard deviations of each condition were excluded step by step. Then, medians were calculated for each condition. Relative to no cue condition, double cues tend to alert the participants to the imminent appearance of the target in the two potential target locations. Accordingly, the alerting effect was calculated by subtracting the mean of medians under the double cue condition from the mean under medians of the no cue condition. Both spatial cues and central cues provide alerting information for the imminent appearance of the target, but only the spatial cues provide predictive spatial information about the appropriate location begin the target arrives. Thus, the orienting effect was calculated by subtracting the mean of medians under the spatial cue condition from the mean of medians under the center cue condition. Regarding target conditions, participants had to overcome the conflict elicited by the distracting flankers in the incongruent condition, whereas they did not in congruent condition. The executive effect was calculated by subtracting the mean of medians under the congruent condition from the mean of medians under the incongruent condition.

### Statistics

Pearson’s Chi squared test was carried out to assess the sex ratio between groups. Multivariate analysis of variance (MANOVA) was performed for index age and educational level between HIV-infected patients and normal controls.

To provide a comprehensive description of the results, we first conducted a three-way repeated analysis of variance (repeated measures ANOVA) with groups (HIV-infected patients vs. normal controls) as a between-subjects factor and cue conditions (no cue, center cue, double cue, and spatial cue), and target conditions (neutral, congruent, and incongruent) as within-subject factors, and with response time and accuracy as dependent variables. To clarify which conditions are driving the difference of three attentional network effects between HIV-infected patients and normal controls, we conducted repeated measures ANOVAs with the medians of response time as dependent variables to explored main effects of alerting cue conditions (i.e., double cue vs. no cue), orienting cue conditions (i.e., spatial cue vs. center cue), and executive target conditions (i.e., incongruent target vs. congruent target), and its interaction with groups.

Finally, to explore the possible association between a nadir CD4 T cell count and the pattern of attention network function, MANOVA with post hoc test by Bonferroni was conducted for three attentional network effects, mean RT, and overall accuracy among groups (i.e., patients with CD4 ≤ 200, patients with CD4 > 200, and normal controls).

## Results

### Demographic data

Table 1 summarizes the demographic characteristics between the patients and controls. Analyses of Variance and Chi Squared tests revealed no significant differences in index age, education levels, and sex ratio.

### Repeated measures ANOVA for RT

Table 2 summarized the performance in ANT for each group. Repeated measures ANOVA results showed a significant main effect of cue conditions (i.e., longer RT in no cue condition than others, and shorter RT in spatial condition than others) [*F* (3168) = 131.757, *P* < 0.001]. There was significant main effect of target conditions (i.e., longer RT in incongruent than others and shorter RT in neutral) [*F* (2112) = 447.633, *P* < 0.001]. No significant main effect of group was found for RT [*F* (156) = 0.016, *P* = 0.899].

**Table 2:** Mean RT and accuracy under each condition for each group

	No cue		Double cue		Center cue		Spatial cue		Mean	
	Normal controls	HIV-infected patients	Normal controls	HIV-infected patients	Normal controls	HIV-infected patients	Normal controls	HIV-infected patients	Normal controls	HIV-infected patients
Mean RT and standard deviations										
Congruent	709 (123)	695 (99)	653 (104)	668 (83)	673 (100)	668 (94)	640 (105)	630 (93)	669 (108)	666 (95)
Incongruent	792 (121)	802 (85)	768 (110)	782 (83)	773 (104)	791 (83)	723 (106)	747 (88)	764 (110)	781 (85)
Neutral	634 (118)	619 (101)	574 (89)	585 (85)	586 (101)	596 (87)	569 (93)	570 (88)	591 (100)	593 (90)
Mean	712 (121)	706 (95)	665 (101)	678 (87)	677 (101)	685 (88)	644 (101)	649 (89)	674 (106)	679 (90)
Accuracy and standard deviations										
Congruent	0.98 (0.07)	0.99 (0.04)	0.97 (0.07)	1.00 (0.02)	0.98 (0.07)	0.99 (0.06)	0.98 (0.07)	0.99 (0.03)	0.98 (0.07)	0.99 (0.04)
Incongruent	0.96 (0.08)	0.98 (0.04)	0.95 (0.08)	0.98 (0.05)	0.94 (0.09)	0.97 (0.06)	0.95 (0.08)	0.98 (0.06)	0.95 (0.08)	0.98 (0.05)
Neutral	0.97 (0.08)	0.98 (0.08)	0.97 (0.07)	0.98 (0.03)	0.98 (0.07)	0.98 (0.05)	0.97 (0.08)	0.98 (0.05)	0.97 (0.07)	0.98 (0.06)
Mean	0.97 (0.07)	0.98 (0.06)	0.97 (0.07)	0.99 (0.04)	0.96 (0.08)	0.98 (0.05)	0.97 (0.08)	0.98 (0.05)	0.97 (0.08)	0.98 (0.05)

**Fig. 2:**
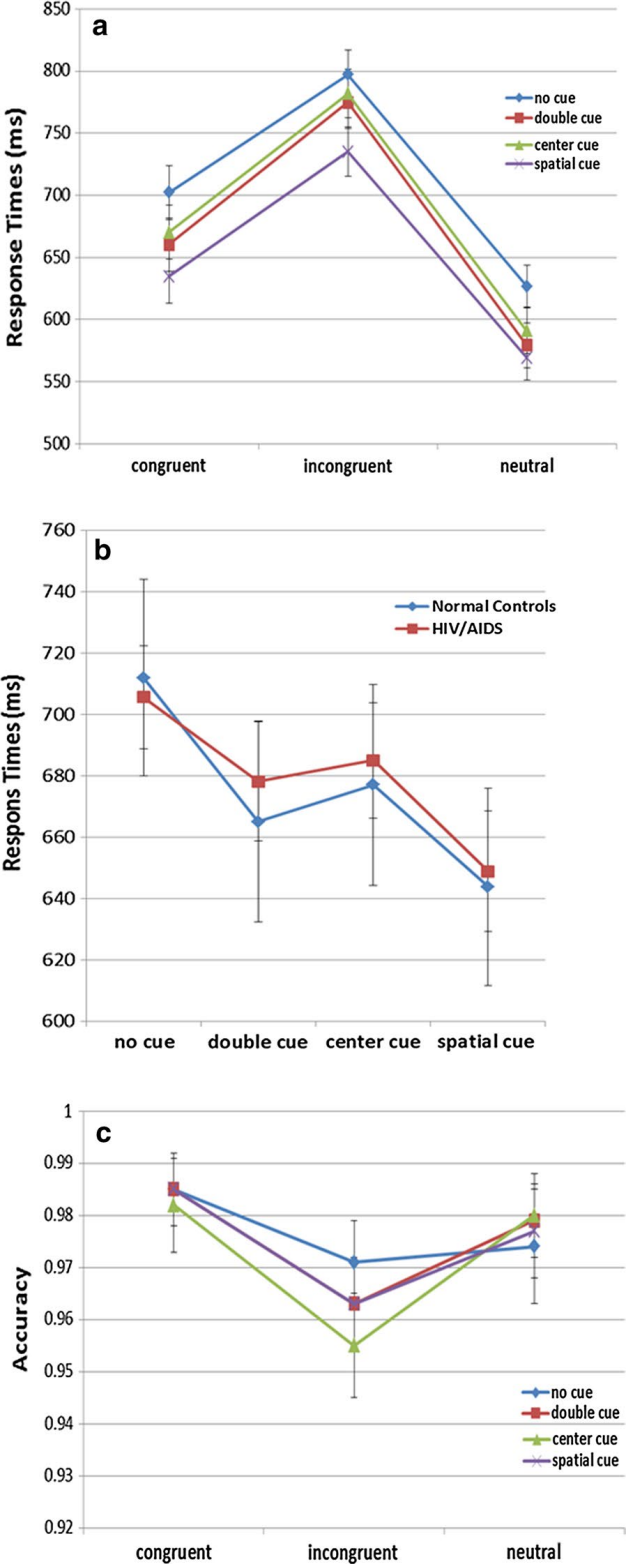
The diagram of the significant interactions between different conditions for RT. No significant interaction between target conditions and group was found [*F* (2112) = 1.442, *P* = 0.241]. No significant interaction between cue conditions, target conditions and group was found [*F χ*(6336) = 1.645, *P* = 0.134]

There was a significant interaction between cue conditions and target conditions [*F* (6336) = 5.848, *P* < 0.001]. As Fig. 2a shows, the effect of the target conditions was enhanced when given double cues and center cues than that in no cue condition and spatial cue condition. Additionally, there was a significant interaction between cue conditions and group [*F* (3168) = 3.362, *P* = 0.020]. As Fig. 2b shows, participants in group of HIV-infected showed longer RT in double cue condition than normal controls, but approximately equal RT with normal controls in no cue condition and spatial cue condition.

### Repeated measures ANOVA for accuracy

Repeated measures ANOVA results showed a significant main effect of target conditions (i.e., participants made more errors in incongruent condition than others) [*F* (2112) = 10.876, *P* < 0.001]. The interaction between cue conditions and target conditions approached significance [*F* (6336) = 2.225, *P* = 0.040]. As Fig. 2c shows, the effect of cue conditions was enhanced when given incongruent target than that in others. No other significant main effects and interactions were found for accuracy.

### Repeated measures ANOVA for medians of response time between normal controls and HIV-infected patients

Repeated measures ANOVA for medians of response time showed significant main effects of alerting cue conditions (i.e., longer medians in no cue than double cue) [*F* (156) = 146.386, *P* < 0.001], orienting cue conditions (i.e., longer medians in center cue than spatial cue) [*F* (156) = 102.671, *P* < 0.001], and executive target conditions (i.e., longer medians in incongruent target than congruent target) [*F* (156) = 102.671, *P* < 0.001] [*F* (156) = 430.346, *P* < 0.001].

**Fig. 3:**
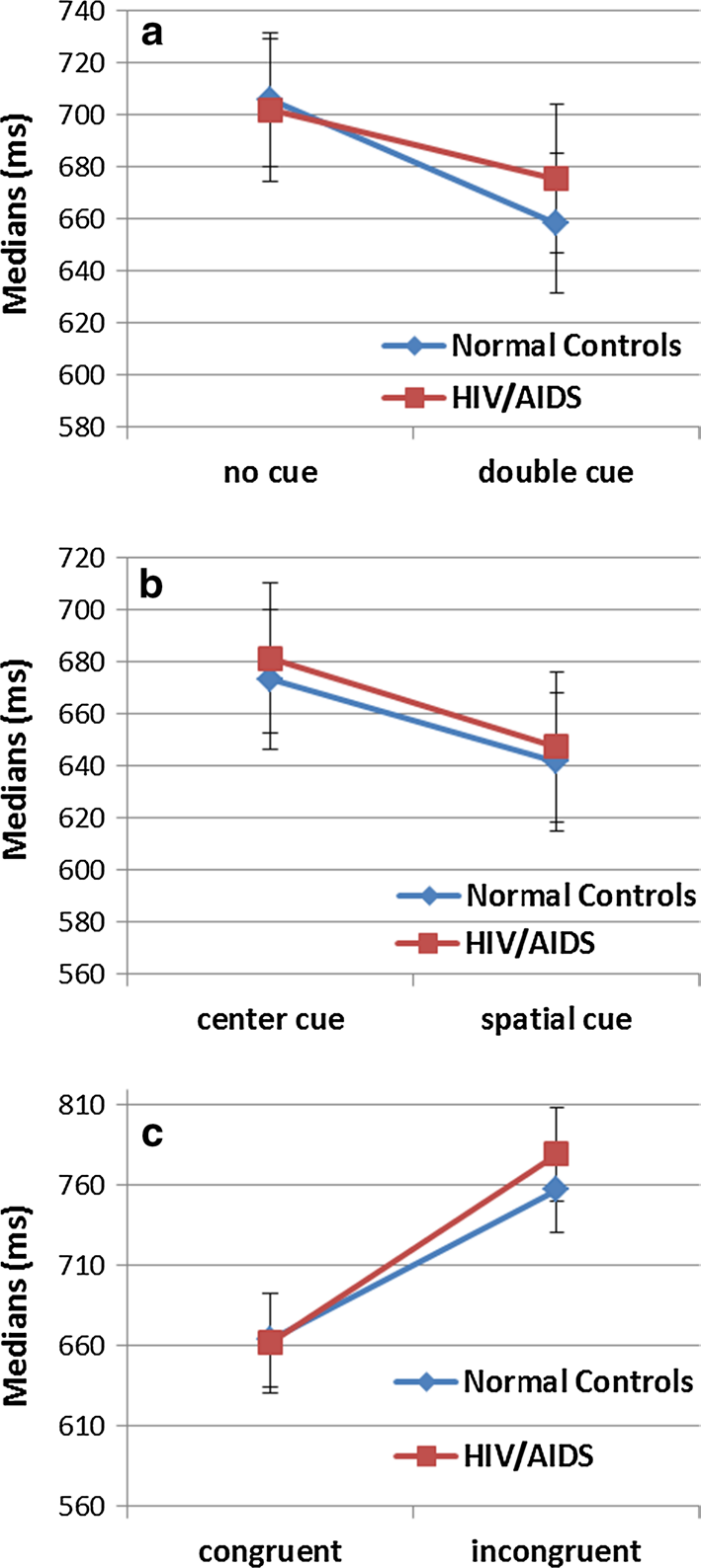
The diagram of the interactions between different conditions for medians of RT

There was a significant interaction between alerting cue conditions and groups [*F* (156) = 11.948, *P* = 0.001]. As Fig. 3a shows, participants in group of HIV-infected showed longer medians in double cue condition than normal controls, but approximately equal medians with normal controls in no cue condition. No significant interaction was found between orienting cue conditions and groups [*F* (156) = 0.143, *P* = 0.707]. As Fig. 3b shows, both HIV-infected patients and normal controls showed longer medians in center cue condition than spatial cue condition. Repeated measures ANOVA also indicated a significant interaction between executive target conditions and groups [*F* (156) = 5.800, *P* = 0.019]. As Fig. 3c shows, participants in group of HIV-infected showed longer medians in incongruent target condition than normal controls, but approximately equal medians with normal controls in congruent target condition.

### Attentional network effects among groups

Table 3 summarized three attentional network effects for each group. MANOVA results showed that there was a significant main effect of groups on alerting effect [*F* (255) = 7.261, *P* = 0.002] and executive effect [*F* (255) = 3.208, *P* = 0.048], but not on orienting effect [*F* (255) = 1.348, *P* = 0.268]. No significant differences were found between groups for mean RT [*F* (255) = 1.236, *P* = 0.298] and overall accuracy [*F* (255) = 0.761, *P* = 0.472].

**Table 3:** Comparisons of ANT performance between groups (Mean ± SE)

	Normal controls (n = 31)	HIV-infected patients (n = 27)			Statistics
		CD4 > 200 (n = 12)	CD4 ≤ 200 (n = 15)	Overall	
Alerting effect (ms)	47.06 ± 3.73	32.27 ± 5.88	18.58 ± 6.58	26.19 ± 4.90	*F* = 7.261, *p* = 0.002
Orienting effect (ms)	31.35 ± 4.02	40.40 ± 6.25	25.42 ± 6.99	33.74 ± 5.16	*F* = 1.348, *p* = 0.268
Executive effect (ms)	92.84 ± 4.92	111.60 ± 9.99	124.17 ± 11.17	117.19 ± 9.32	*F* = 3.208, *p* = 0.048
Mean RT (ms)	669.19 ± 31.97	636.87 ± 37.35	724.42 ± 41.75	675.78 ± 18.90	*F* = 1.236, *p* = 0.298
Overall accuracy (%)	96.16 ± 1.23	97.33 ± 1.53	98.58 ± 1.71	97.89 ± 4.49	*F* = 0.761, *p* = 0.472

Post-hoc tests by Bonferroni showed that the patients with CD4 ≤ 200 exhibited less efficient alerting attention than normal controls (*P* = 0.002). The difference between the patients with CD4 ≤ 200 and normal controls approached significance (*P* = 0.062). No other significances were found between-groups comparisons (all *P* ≥ 0.347).

## Discussion

Attention is the core cognitive ability to select some aspects of the world and ignore others for further processing [11, 29]. While this basic ability enables the acquisition of skills in other cognitive areas, few studies in HIV-infected adults have employed measures specifically designed to assess different components of attentional ability. For its advantage of allowing for comparison of the relative deficits in different attention network [20], the attention network test (ANT) has been widely used in different neuropsychological population. In the present work, we first adopted ANT to test whether the HIV-infected patients showed a specific attention network deficit or a general attentional impairment.

Our results show that HIV-infected patients exhibited longer medians in double cue condition than normal controls, whereas made approximately equal medians with normal controls in no cue condition. This result indicate that HIV-infected patients showed less advantage from a warning cue for preparation for detecting an expected signal, and suggest that they exhibited less efficient alerting attention function. This finding is consistent with the neurotransmitter hypothesis underlying alerting attention. It is believed that the alerting attention is related to norepinephrine function [18]. Patients with HIV-infection often complained about their difficulty in maintaining a vigilant state [15, 30]. And, previous studies have reported that the norepinephrine response to a cold pressor test [31] and the sympathetic activity [32] in HIV-infected subjects was blunted. Our result also indicate HIV-infected patients showed longer medians in incongruent target condition than normal controls, while made approximately equal medians with normal controls in congruent target condition. These findings suggest that patients with HIV-infection exhibited less efficient executive attention function, and had difficulty in resolving response conflicts between competing information. This is consistent with previous neuropsychological studies in HIV-infection. Deficits in executive domain of attention control have been reported in previous studies using the trail making test [33], and the Stroop task [34]. Also, this is in agreement with the neurotransmitter hypothesis underlying executive attention. It is demonstrated that the dopamine function plays a prominent role in executive attention network [18]. And, previous studies have reported that the cerebrospinal fluid dopamine mean values were significantly lower in HIV-infected subjects than HIV-seronegative group [35, 36]. In addition, Kumar et al. reported a strong correlation between specific HIV-1 viral load increases and dopamine reduction within the Substantia Nigra [37].

Unlike the findings of alerting attention and executive attention, our results indicate that HIV-infected patients were equally sensitive to the spatial cues with normal controls. These seem to suggest that the orienting attention function was intact in these patients. The intact orienting attention function found in HIV-infected patients is not unique. Using ANT, we have also found that an intact orienting attentional ability in patients with depression [38], untreated hyperthyroidism [39], and the breast cancer patients receiving chemotherapy treatment [40]. Previous studies have documented that cholinergic systems arising in the basal forebrain appear to play a critical role in orienting attention network [18, 41, 42]. Further research should be conducted to examine whether this cholinergic systems are not vulnerable to HIV infection.

Consistent with previous literature [43–46], our results also indicate a tendency for poorer efficiency on alerting attention and executive attention in patients with CD4 ≤ 200. Although this finding was limited by small sample size, the link between lower CD4 T cell count and poorer attention network function has revealed the importance of the CD4 nadir as a marker of neurocognitive deficits. As Muñoz-Moreno et al. [46] proposed, neurocognitive functioning is likely to be more affected when more systemic immunosuppression appears. In addition, a recent study has documented those reductions in brain volumes in HIV-infected patients are strongly linked to a history of immunosuppression with lower nadir CD4 count [47]. This finding, combined with other evidences, suggests that it would be wiser to start antiretroviral therapy earlier to avoid irreversible neurocognitive impairment.

Some limitations with regard to the present study should be mentioned. For absence of day-to-day functioning scores in HIV-infected patients, it is not known whether there is an association between everyday functioning and the three attention network effects. The lack of clinically-relevant information about HIV disease prognosis also makes it difficult to interpret these findings within the context of HIV disease. Finally, our sample size was small, which increases the risk of type II error. In sum, although limited by a small sample size, our findings suggest that HIV-infected patients exhibited selective impairments of attention network of alerting and executive control. It would be wiser to start antiretroviral therapy earlier to avoid irreversible neurocognitive impairment.

## Conclusions

The use of ANT allowed us to address whether the attentional deficits in HIV-infected patients was a specific attention network deficit or a general attention deficit. Our findings indicated that HIV-infected exhibited selective impairments of attention network of alerting and executive control, whereas orienting attention was intact. Our results also indicate a tendency for poorer efficiency on alerting attention and executive attention in patients with CD4 ≤ 200. Despite of several limitations in the present work, our results will be helpful in providing a better understanding of attentional deficits in HIV-infected patients.
